# Placenta Derived Mesenchymal Stem Cells Hosted on RKKP Glass-Ceramic: A Tissue Engineering Strategy for Bone Regenerative Medicine Applications

**DOI:** 10.1155/2016/3657906

**Published:** 2016-12-19

**Authors:** Mario Ledda, Marco Fosca, Angela De Bonis, Mariangela Curcio, Roberto Teghil, Maria Grazia Lolli, Adriana De Stefanis, Rodolfo Marchese, Julietta V. Rau, Antonella Lisi

**Affiliations:** ^1^Institute of Translational Pharmacology (IFT-CNR), Via del Fosso del Cavaliere 100, 00133 Rome, Italy; ^2^Istituto di Struttura della Materia (ISM-CNR), Via del Fosso del Cavaliere 100, 00133 Rome, Italy; ^3^Dipartimento di Scienze, Università della Basilicata, Via dell'Ateneo Lucano 10, 85100 Potenza, Italy; ^4^Istituto di Struttura della Materia (ISM-CNR), Montelibretti Unit, Via Salaria km 29.300, 00015 Monterotondo Scalo, Italy; ^5^S. Peter Hospital FBF, Rome, Italy

## Abstract

In tissue engineering protocols, the survival of transplanted stem cells is a limiting factor that could be overcome using a cell delivery matrix able to support cell proliferation and differentiation. With this aim, we studied the cell-friendly and biocompatible behavior of RKKP glass-ceramic coated Titanium (Ti) surface seeded with human amniotic mesenchymal stromal cells (hAMSCs) from placenta. The sol-gel synthesis procedure was used to prepare the RKKP glass-ceramic material, which was then deposited onto the Ti surface by Pulsed Laser Deposition method. The cell metabolic activity and proliferation rate, the cytoskeletal actin organization, and the cell cycle phase distribution in hAMSCs seeded on the RKKP coated Ti surface revealed no significant differences when compared to the cells grown on the treated plastic Petri dish. The health of of hAMSCs was also analysed studying the mRNA expressions of MSC key genes and the osteogenic commitment capability using qRT-PCR analysis which resulted in being unchanged in both substrates. In this study, the combination of the hAMSCs' properties together with the bioactive characteristics of RKKP glass-ceramics was investigated and the results obtained indicate its possible use as a new and interesting cell delivery system for bone tissue engineering and regenerative medicine applications.

## 1. Introduction

Multipotent mesenchymal stem cells (MSCs) are a promising source for tissue regeneration thanks to their ability of self-renewal and capability of differentiating into various cell lineages including chondrocytes, osteoblasts, and adipocytes. MSCs reside in several human tissues and in the case of injury, they play an important role in the processes of tissue repair through the secretion of trophic factors that may act directly triggering the intracellular mechanisms of injured cells, or indirectly inducing secretion of functionally active mediators from the neighboring cells [1–3].

The initial approaches in the use of MSCs for bone tissue engineering have shown encouraging results in animal models [4–6] and in human patients [7, 8]. Nevertheless, after human MSC transplantation, patients must be treated with high doses of growth factors necessary for bone formation, but this treatment highlights negative side effects in many patients [9, 10].

MSCs, which were originally isolated from bone marrow, were later obtained also from other sources, including amniotic fluid and placenta. Human amniotic mesenchymal stromal cells (hAMSCs), derived from the amniotic fetal membrane of human term placenta, have generated great interest in the scientific community for their immunomodulatory and proregenerative properties. Their use avoids many ethical issues as placenta is generally discarded after birth, they are available in large supplies, and their isolation is not invasive for the donor [11–13]. Altogether these features render hAMSCs an excellent candidate for applications in cell therapy and regenerative medicine protocols [14–17].

Regarding cell delivery, a commonly used approach in cell therapy is to suspend stem cells in a buffer and inject them into the damaged tissue; however this procedure has not shown a satisfactory engraftment rate [6].

For this reason and due to the limited survival of stem cells suspended in a buffer, the engraftment rate may be improved by using a supporting matrix such as a scaffold or a hydrogel [18]. An ideal biomaterial scaffold for cell delivery supporting osteogenesis has not been yet identified [19], and current efforts are directed towards the design of a scaffold able to heal bone defects in specific anatomic sites and also favorably affect bone formation by stimulating osteoblastic cell proliferation and differentiation [20].

The present trends in biomaterials science aim to develop properly engineered porous three-dimensional scaffolds, possessing necessary mechanical characteristics, able to replace, repair, and regenerate damaged tissues favouring cell adhesion, growth, and differentiation. Various materials are employed for this scope, such as bioactive ceramics and glasses, biodegradable polymers, and their composites [21, 22]. Moreover, the current challenge in biomaterials design is to match the kinetics between the biomaterial's degradation and the newly formed tissue, triggering and stimulating the effective development of new tissue growth. In this context, bioactive glasses represent a promising biodegradable material type to be used for bone tissue engineering [23–25]. Bioactive glasses have unique properties, one of them being the ability to form a carbonated hydroxyapatite layer when exposed to biological fluids, a layer responsible for the strong binding between bioactive glasses and host tissue [23, 26].

There are many compositional groups of bioactive glasses, each with its own specialization. The biological response from bioactive glasses was studied in detail [20] and recently, their gene activating properties, due to the release of ionic products in solution, were discovered stressing their remarkable ability to stimulate gene expression [27]. In particular, this process is able to stimulate the “in vitro” and “in vivo” expression of several osteoblastic genes [20, 28] and the angiogenesis process [29, 30].

Two methods are currently available to produce bioactive glasses: high temperature melt-processing and low temperature sol-gel method. This later procedure is a much more versatile tool, especially for what concerns compositional variation and nanoscale additions. We have reported the application of a sol-gel synthesis procedure to prepare the RKKP glass-ceramic material [31]. This material, developed at ISTEC-CNR (Faenza, Italy), was obtained adding a small amount of Ta_2_O_5_ and La_2_O_3_ oxides to the AP40 composition. The La^3+^/Ta^5+^ ions couple was found to be crucial in supplying the biomaterial surface with a suitable Z potential which is able to regulate the adherence of proteins [32, 33].

Titanium is widely applied in orthopaedics and dentistry due to its well-known good biocompatibility with hard and soft tissues. Furthermore, due to its outstanding mechanical properties, it is widely used for hard tissue implants, especially for load-bearing applications. Nevertheless, it is oxidised in the presence of body fluids surrounding the implant. This leads to the formation of Ti oxides and debris accumulation, occasionally leading to the implant loss. Therefore, in order to improve wear resistance, the Ti surface is coated with various materials including RKKP glass-ceramics, and in a recent publication we studied the CaCo-2 cell-friendly properties of the RKKP coated Ti surface, demonstrating that it represents a suitable substrate for cells adhesion and growth [34].

In this paper, for the first time we report the effects of the RKKP coated Ti support on human amniotic mesenchymal stem cells' growth, proliferation, metabolic activity, cell morphology, cycle phase distribution, and mRNA expressions of key genes including osteogenic differentiation markers.

## 2. Materials and Methods

### 2.1. Preparation of RKKP Glass-Ceramic Granules by Sol-Gel Synthesis

The synthesis was performed according to details given in [31, 35]. The final glass-ceramics, obtained by sol-gel, had the following composition [13]: SiO_2_ [43.68], P_2_O_5_ [11.10], CaO [31.30], Na_2_O [4.53], MgO [2.78], CaF_2_ [4.92], K_2_O [0.19], La_2_O_3_ [0.50], and Ta_2_O_5_ [1.00]. An aqueous solution of TEOS (tetraethyl orthosilicate), P(OEt)_3_, Ca(NO_3_)_2_·4H_2_O, NaNO_3_, Mg(NO_3_)_2_·6H_2_O, KNO_3_, NH_4_F, La(NO_3_)_3_·6H_2_O, and Ta(OC_2_H_5_)_5_, balanced in stoichiometric amounts, underwent hydrolysis and polycondensation to obtain the desired composition. To catalyse TEOS and P(OEt)_3_ hydrolysis, HNO_3_ (0.1 M) was utilised. All products were from Sigma-Aldrich, >99.9% purity, and were used as received. The synthesis was carried out in a Teflon bottle. Reactants were added to the mixture one by one, under vigorous stirring, every 30 minutes. The addition followed the order: TEOS, triethyl phosphite, calcium nitrate tetrahydrate, sodium nitrate, magnesium nitrate hexahydrate, ammonium fluoride, potassium nitrate, lanthanum nitrate hexahydrate, and tantalum ethoxide. The sol was left at room temperature for 10 days and then placed in an oven at 70°C for 72 h to obtain a gel. This gel was dried at 120°C for 48 h. It was then stabilized at 700°C (heating rate 5°C/min, cooling rate 20°C/min) to obtain sol-gel granules, which were pressed at 400 MPa and sintered in air at a peak temperature of 1100°C (heating rate 5°C/min, cooling rate 20°C/min).

X-Ray powder diffraction patterns were obtained on a Philips PW 1130 diffractometer using Ni-filtered Cu Ka radiation (*λ* = 1.5418 Å).

### 2.2. Pulsed Laser Deposition of Films

From the so-obtained pressed and sintered granules, a RKKP tablet-target was prepared and subsequently used for deposition of films. The deposition experiments were carried out applying a Pulsed Laser Deposition (PLD) technique. PLD experimental setup consists of a stainless steel vacuum chamber, evacuated to a pressure of 1.5 × 10^−4^ Pa, supplied by a rotating target holder and a heated substrate support. The ablation laser source was a Quanta System frequency doubled Nd:YAG laser (emission wavelength 532 nm, repetition rate 10 Hz, and pulse duration 10 ns). Depositions were performed under the following conditions: 12 J/cm^2^ of laser fluence; 45° laser beam incidence with respect to the target surface; substrate kept at 2 cm from the target; deposition time 2 h. All the films were deposited at a substrate temperature of 500°C. Pure Ti was used as substrate material (1 × 1 cm^2^). Ti substrates were sandblasted with a 60-grid SiC abrasive powder in order to assure nanometric surface roughness. In order to remove any contaminant from the surface, the substrates were boiled in aqua regia for 30 min.

### 2.3. Characterisation Techniques

Several characterization techniques were used to test coating's properties, namely, Energy Dispersive X-Ray Diffraction (EDXRD), Atomic Force Microscopy (AFM), and Scanning Electron Microscopy, coupled with Energy Dispersive X-ray Spectroscopy analysis (SEM-EDS).

For EDXRD measurements, a noncommercial ED apparatus was utilised, consisting of two arms pivoting around the optical centre. sample holder. White X-ray radiation is produced by a commercial W-anode X-ray tube (12–55 keV) and is collimated upstream and downhill the sample by four W-slits. X-ray diffraction pattern represents in this case the intensity of the X-ray radiation elastically scattered by a sample as a function of the momentum transfer. The momentum transfer amplitude takes the name of scattering parameter: *q*(*E*, *θ*) = *aE*sin⁡*θ*, *E* being the electromagnetic radiation energy, 2*θ*, the scattering angle, and *a*, a constant (1.014 Å/keV). The coatings were investigated in reflection mode in order to minimize the absorption contribution arising from the Ti polycrystalline substrate and to maximize the film-to-substrate diffracted intensity ratio.

A noncommercial Atomic Force Microscope, working in the noncontact modality, was applied to investigate the surface morphology. High-resolution (5 × 5 *µ*m^2^) topographies were recorded with a precision limit of about 0.1 nm.

SEM (LEO 1450 Variable Pressure), coupled with EDS (INCA Energy 300 detector), was used to investigate the elemental composition of films and bulk RKKP material.

### 2.4. hAMSC Isolation and Culture

According to the approved protocol N°64/2012/C.B. from the Ethical Committee of the FBF S. Peter Hospital, we obtained human full-term placentas from healthy women after written informed consent. In general, placentas were processed immediately and hAMSCs isolated from the amniotic membrane dissected from the deflected part of the fetal membranes to minimize the presence of maternal cells. All the experiments were performed seeding the hAMSCs from passages P1 up to P4 (1 × 10^4^ cells/cm^2^) either on the RKKP coated Ti surface or on a treated commercial plastic Petri dish (as a control sample, CTR) and grown up to 4 days. The RKKP coated Ti surfaces were sterilized for 1 hour using an ethanol alcohol treatment and then washed three times with phosphate-buffered saline (PBS). The cells (10 × 10^4^ cells/cm^2^) were also seeded on the noncoated Ti substrate. The protocols used for hAMSC isolation and culture are better described in the Supplementary Materials section in Supplementary Material available online at http://dx.doi.org/10.1155/2016/3657906.

### 2.5. Cell Proliferation and Metabolic Activity Analysis

The hAMSC proliferation was evaluated by Bromodeoxyuridine incorporation assay (BrdU, Cell Proliferation Kit, Roche Diagnostics). The hAMSC metabolic activity was quantified by a colorimetric assay based on the oxidation of water-soluble tetrazolium salts (Cell Proliferation Reagent WST-1, Roche Diagnostics). The protocols used are better described in the Supplementary Materials section.

### 2.6. Real-Time Quantitative RT-PCR Analysis

Total RNA was extracted from hAMSCs grown on both the treated plastic Petri dish and the RKKP film surface for 4 days using TRIzol Reagent (Invitrogen). One microgram of total RNA was used to synthesize first strand cDNA with random primers, using 100 U of ImProm-II RT-PCR kit (Promega, Madison, WI, USA). The quantification of all gene transcripts was carried out by real-time quantitative reverse transcriptase polymerase chain reaction (RT-PCR). The protocols used are better described in the Supplementary Materials section and the primers used are shown in Table 1.

### 2.7. Cytoskeleton Analysis by Actin Fluorescence Staining

The hAMSCs were seeded both on the RKKP coated Ti surface and on the treated plastic Petri dish at a density of 1 × 10^4^ cells/cm^2^ and cultured for 4 days. Cells were washed in phosphate-buffered saline (PBS), fixed in paraformaldehyde 4% in PBS for 15 min, and incubated with phalloidin tetramethylrhodamine isothiocyanate-conjugate (1 : 500), an anti-actin toxin (Sigma), in a PBS buffer for 1 h. Cells were washed three times with PBS, counterstained for nuclei localization with Hoechst 33342 (trihydrochloride-trihydrate), and examined. The RKKP coated Ti surfaces were overturned on cover glasses and tested by direct fluorescence for the presence of actin filaments. Fluorescence measurements were obtained using an inverted microscope (Olympus IX51, RT Slider SPOT, Diagnostic instruments, Sterling Heights, MI, USA), equipped with a 20 X objective and with a cooled CCD camera (Spot RT Slider, Diagnostic Instruments).

### 2.8. Cell Cycle and Immunophenotypic Analysis by Flow Cytometry

The hAMSCs were grown both on the RKKP coated Ti surface and on the treated plastic Petri dish. After two days of culture, the cells were detached with trypsin and washed in cold FACS buffer (2 mM EDTA, 0.5% FBS in PBS 1x) in order to obtain a cell suspension without cell clumps. For the cell cycle analysis, the cells were washed twice in PBS, suspended in 1 mL PBS, and then fixed in 10 mL 70% cold ethanol at 4°C. Fixed cells were washed in PBS and then stained with propidium iodide (20 ug/mL, Sigma) and RNase A (250 ug/mL, Sigma) solution for 30 min at room temperature in the dark. About 2 × 10^5^ cells were acquired using a FACSCalibur (Becton Dickinson) cytometer and the cell cycle analysis was performed by ModFIT LT 2.0 software.

For the immunophenotypic analysis, the cells were incubated for 30 minutes on ice with mouse monoclonal antibodies to human CD29-FITC (HI29a, Immunotools Friesoythe, Germany), CD73-PE (AD2, BioLegend), and CD31-APC (MEM-05, Immunotools). Isotype control antibodies IgG1-FITC, IgG1, k-PE, and IgG1-APC (Immunotools) were used for background normalization. Dead cells exclusion was performed adding 5 ug/mL propidium iodide to the samples prior to analysis. About 2 × 10^5^ cells were acquired using a FACSCalibur (Becton Dickinson) cytometer and data were analysed by Cell Quest Pro software (Becton Dickinson).

### 2.9. In Vitro Osteogenic Commitment Study

The hAMSCs were seeded both on the RKKP coated Ti surface and on the treated plastic Petri dish at a density of 1 × 10^4^ cells/cm^2^ and cultured in DMEM (HG) (Sigma) supplemented with 10% FBS, 10 mM *β*-glycerophosphate, 0.2 mM ascorbic acid, and 10^−8^ M dexamethasone (Sigma), for 1 week (Osteogenic Medium, OM). The osteogenic commitment was evaluated by real-time RT-PCR to analyse the mRNA expression of differentiation markers: RUNX2, Osteocalcin (OCL), and Alkaline Phosphatase (ALP).

### 2.10. Statistics Analysis

The statistical analysis of the data obtained was performed using Student's *t*-test, with *p* < 0.05 as the minimum level of significance.

## 3. Results

### 3.1. RKKP Coated Ti Supports

**Figure 1:**
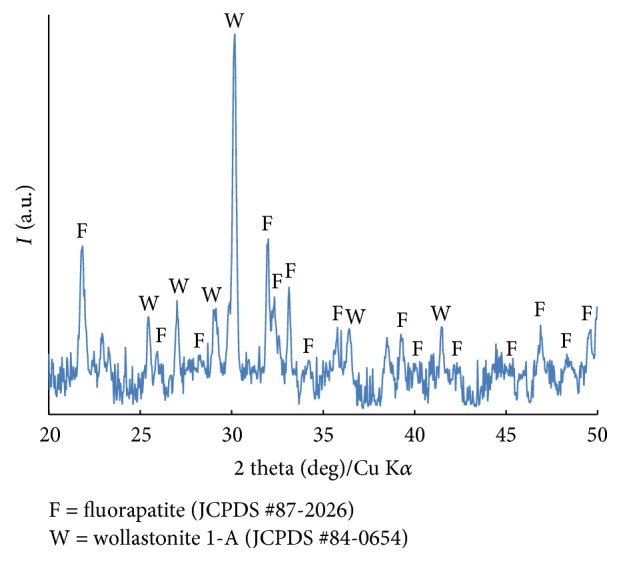
XRD spectrum of sol-gel RKKP bulk target material.

**Figure 2:**
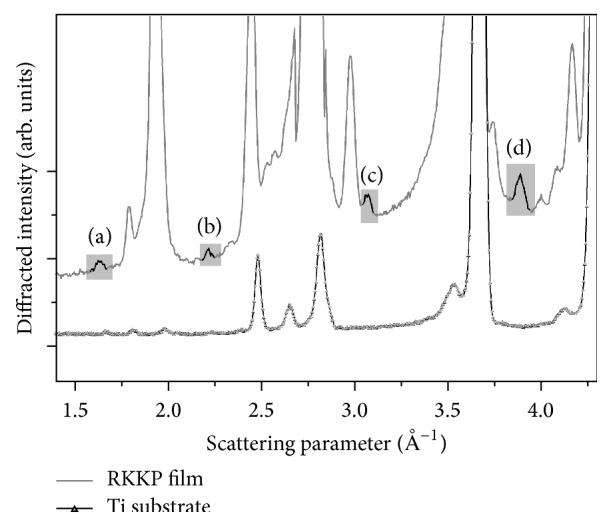
EDXRD spectra of deposited RKKP film and Ti substrate.

The physicochemical characterisation of the RKKP bulk target material and films on Ti was studied [31, 35]. In this work, the XRD pattern of the synthesized RKKP material (Figure 1) and the EDXRD patterns of the RKKP film and Ti substrate are reported (Figure 2). As it can be observed, due to the intense Ti substrate peak contributions, it is difficult to distinguish the film pattern. Only a few peaks, attributable to RKKP phase composition, were identified (see reflections (a)–(d) in Figure 2 at (a) *q* = 1.6 Å; (b) *q* = 2.2 Å; (c) *q* = 3.0 Å; and (d) *q* = 3.9 Å). The (a) reflection can be attributed to the CaSiO_3_ phase, whereas the other peaks have contributions of several phases, that is, (b) *β*-Ca_3_(PO_4_)_2_ and fluorinated hydroxyapatite (F-HAp); (c) *β*-Ca_3_(PO_4_)_2_ and CaSiO_3_; and (d) *β*-Ca_3_(PO_4_)_2_, F-HAp, and CaSiO_3_. For further characterization, SEM-EDS elemental analysis was carried out, and the data obtained are presented in Table 2. As it can be seen, there is a good correlation between the target and the deposited film's composition.

**Figure 3:**
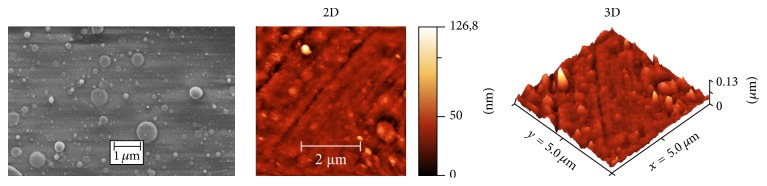
SEM and AFM (2D and 3D) images of RKKP film surface.

In Figure 3, surface characterization results are presented. On the left part of Figure 3, the SEM image of the surface morphology of the RKKP coated Ti supports is shown. As it can be seen, the surface is dense and compact, with the presence of some submicrometric droplets, characteristic for PLD deposition [36] The film's thickness, measured from the cross-sectional SEM images, resulted to be about 4.0 ± 0.5 *µ*m.

For a more detailed surface topography characterization, AFM measurements were performed. In Figure 3, the AFM 2D and 3D images, both corresponding to 5 × 5 *µ*m^2^ areas, are presented. On the 2D AFM image, *Z* scale dimension is represented in false colours by means of the vertical bar, ranging up to approximately of 127 nm. For better representation, 3D view of the same image is shown on the right part of Figure 3. The same area was considered to analyse the typical surface roughness (r.m.s.). The r.m.s., representing the roughness for the entire scanned area, was calculated to be 8 ± 1 nm. To summarize, the PLD deposited RKKP coating on Ti support has the following characteristics: dense and compact morphology, RKKP composition, thickness of 4.0 ± 0.5 *µ*m, and average surface roughness of 8 ± 1 nm.

### 3.2. Cell Metabolic Activity and Proliferation

**Figure 4:**
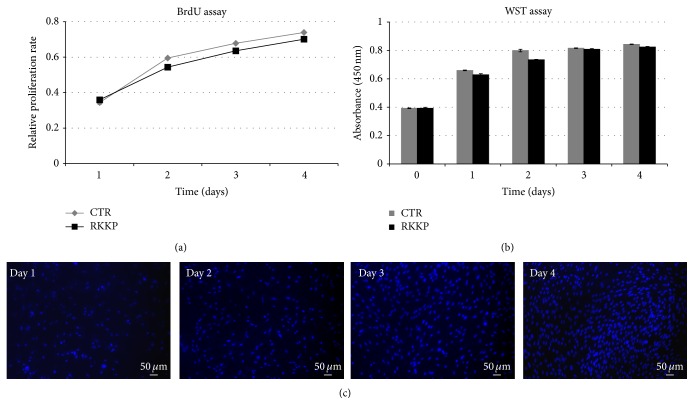
(a) Cell proliferation study by BrdU incorporation assay of hAMSCs, seeded on RKKP coated Titanium surface (RKKP) and on treated plastic Petri dish (CTR). (b) Cell metabolic activity analysis of hAMSCs by WST-1 assay of hAMSCs seeded on RKKP coated Titanium surface (RKKP) and on treated plastic Petri dish (CTR). (c) Time evolution of hAMSCs' nuclei seeded on RKKP coated Titanium surface, revealed by Hoechst staining 33342. Data are shown as mean ± SD.

The hAMSCs were seeded both on the RKKP coated Ti surface and on the treated plastic Petri dish, and their metabolic activity and cell proliferation rate were studied daily by WST-1 colorimetric assay and by BrdU incorporation assay, respectively (Figure 4). The cells plated on the RKKP film surface showed an increasing exponential growth trend similar to the one observed in the control cells (Figure 4(a)). As expected, the metabolic activity of the hAMSCs seeded on the RKKP film surface also increased from day 0 up to day 4, and no significant differences were observed when compared to the control cells (Figure 4(b)). These results were confirmed by the nuclei staining with Hoechst 33342, clearly highlighting from day 1 up to day 4 the increase in number of the hAMSCs grown on the RKKP sample (Figure 4(c)) as well as the even distribution on the substrate. We also investigated the adhesion efficiency of the hAMSCs to the noncoated Ti. We observed that the RKKP sample enabled an increase in cell adhesion capability in comparison to the noncoated Ti, on which the hAMSCs resulted in being more loosely attached (data not shown).

### 3.3. Cell Actin Distribution

The cytoskeleton organization in hAMSCs grown up for 4 days on the RKKP coated Ti surface and the treated plastic Petri dish substrate was investigated.

**Figure 5:**
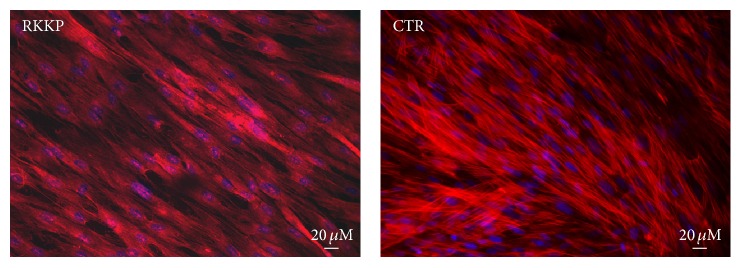
Actin distribution analysis of the hAMSCs seeded on RKKP coated Titanium surface (RKKP) and on treated plastic Petri dish (CTR). 20x magnification.

The hAMSCs were labelled with TRIC conjugated phalloidin and the cytoskeleton distribution was studied. The fluorescence analysis of filamentous actin (F-actin) stained by Phalloidin showed a homogenous distribution of the actin filament organization on the cellular body of both control and RKKP samples (Figure 5).

### 3.4. Cell Cycle Analysis and Immunophenotypical Characterization

The cell cycle phase distribution of hAMSCs, grown for two days on the RKKP coated Ti surface and on the treated Petri dish, was analysed.

**Figure 6:**
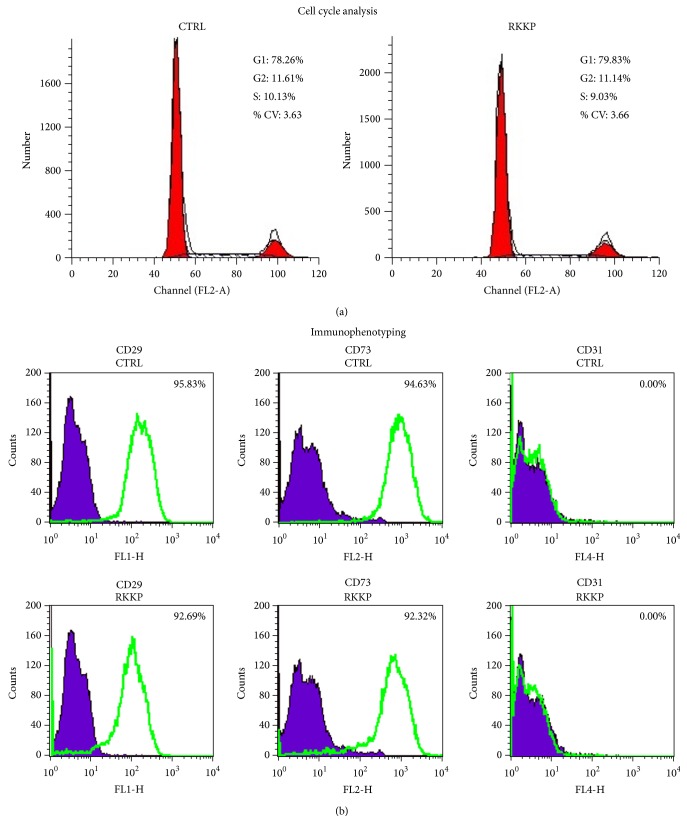
(a) Cell cycle analysis of hAMSCs seeded on RKKP coated Titanium surface (RKKP) and on treated plastic Petri dish (CTR). (b) Immunophenotypical characterization study for CD29, CD73, and CD31 of hAMSCs grown on RKKP coated Titanium surface (RKKP) and on treated plastic Petri dish (CTR).

Single cell suspension of hAMSCs was obtained and the total propidium iodide labelled DNA was studied (Figure 6(a)). We highlighted that the cell phase percentage of the RKKP plated sample detected was 79.63% of cells in G0/G1, 11.14% in S phase, and 9.03% in G2/M and no statistically significant difference was observed in comparison to the control samples (78.26% G0/G1, 11.61% S, and 10.13% G2/M).

The immunophenotypical characterization of hAMSCs, grown for 4 days on the RKKP coated Ti surface and on the treated Petri dish, was also performed (Figure 6(b)). The cells were labelled with antibodies against the mesenchymal CD29 and CD73 stem cell markers and the hematopoietic CD31 stem cell marker and were analysed by flow cytometry. In both samples, the control and the RKKP one, we detected a high CD29 and CD73 cell expression, above 90%, whereas the CD31 resulted in being undetected.

### 3.5. Cell Messenger RNA Expressions

The mRNA expressions of hAMSC key genes grown for 4 days on the RKKP coated Ti surface and on the treated plastic Petri dish were also investigated.

The mRNA transcript levels of anti-inflammatory and trophic factors highly expressed on hAMSCs such as the Transforming Growth Factor beta (TGF*β*), indoleamine 2,3-dioxygenase (IDO), the Vascular Endothelial Growth Factor (VEGF), the Hepatocyte Growth Factor (HGF), and the housekeeping genes (constitutive gene) such as *β*-actin (*β*-ACT), Ki67, and RPL34 were studied.

**Figure 7:**
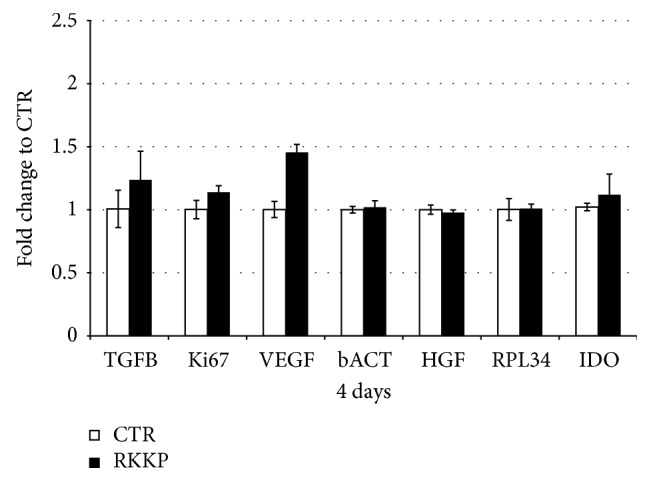
qRT-PCR analysis of hAMSC key genes expressions. The TGF*β*, IDO, VEGF, HGF, and housekeeping genes (constitutive gene) *β*-ACT, Ki67, and RPL34 were investigated in hAMSCs grown on RKKP coated Titanium surface compared to treated plastic Petri dish. Data are shown as mean ± SD.

The messenger expression of these genes in the hAMSCs grown for 4 days on the RKKP film surface resulted in being unchanged when compared to the control sample (Figure 7).

### 3.6. In Vitro Osteogenic Commitment Study

**Figure 8:**
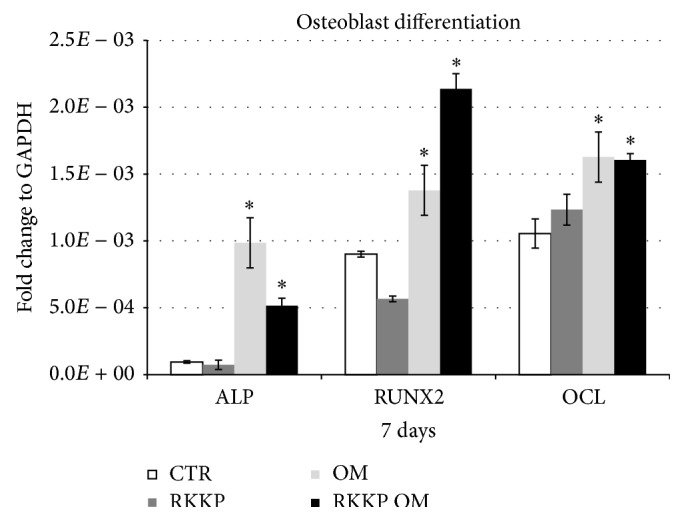
qRT-PCR analysis of the early and late osteoblast differentiation markers (RUNX2, ALP, and OCL) expression on hAMSCs grown on coated Titanium surface compared to treated plastic Petri dish.* Asterisks* identify statistical significance (*p* < 0.05).

The hAMSCs seeded both on the RKKP coated Ti surface and on the treated plastic Petri dish were cultured for up to 7 days in Osteogenic Medium (OM) to verify their osteogenic commitment capability. The mRNA expression study by qRT-PCR assay showed a statistically significant increase of the early differentiation markers such as RUNX2 and Alkaline Phosphatase (ALP), together with an initial upregulation of the late differentiation marker, Osteocalcin (OCL), in the cells grown on both types of substrates (Figure 8).

## 4. Discussion

In the field of regenerative medicine, the development of innovative cell delivery platforms by the combination of cells, scaffolds, and the type and time of the differentiation stimulus induction is very important for the successful building of new tissues.

Osteoporosis is a widespread illness that affects not only postmenopausal women or aged people, but also many patients whose diseases cause osteoporosis directly or as side effect of some therapies [37], developing bone fractures and often requiring implantation of screws and prostheses. These clinical cases could be treated by cellularized biomaterials [38].

The development of biomaterial designs in the emerging field of nanomedicine allows the planning of various nanocomponent additions in order to trigger gene signalling strategies and to form strong bonds with the host living tissues. Among the materials, the bioactive glass composition, 45S5 Bioglass®, discovered by Larry Hench and colleagues in 1969, is being currently used in clinics as a material for implants and regenerative bone-filler for orthopaedic and dental applications [39, 40].

In this framework, we identified the RKKP glass-ceramic, due to its bioactive characteristics, as a suitable material for bone grafting applications in the regenerative medicine protocols [34].

The clinical applications of bioactive glasses depend especially on their intrinsic characteristics and at the same time also on the cell behavior in a certain surrounding environment. For this scope, before the “in vivo” implant, an “in vitro” study is absolutely necessary to test, with appropriate methods, if the synthesized materials are biocompatible and cell-friendly. In this setting, our study tested the “in vitro” effects of RKKP coated Ti surface on the placenta derived mesenchymal stem cells, grown on their surface, and as far as we know, it is the first study performed with this type of stem cells hosted on the RKKP glass-ceramic material.

Firstly, in this work, the biological response of mesenchymal stem cells to this material was tested by checking their cell proliferation rate and metabolic activity daily.

The results of the BrdU incorporation assay showed an increasing exponential growth trend, similar to the one observed in the placenta derived mesenchymal stem cells grown on the treated plastic Petri dishes, our control (Figure 4(a)).

The cell metabolic activity increased and no significant differences were observed when compared to the control cells (Figure 4(b)). The cell attachment, monitored by staining the nuclei, also showed an increase in cell density from day 1 up to day 4 (Figure 4(c)) reaching values near the control level, clearly indicating no cytotoxicity of the RKKP coated Ti surface and suggesting a homogenous surface suitable for the placenta derived mesenchymal stem cells attachment also in terms of surface roughness (of 8 ± 1 nm) (Figure 3).

The cytoskeleton, essential for maintaining cell shape, is involved in a wide variety of cell functions associated with the differentiation process, including the spatial organization of cell organelles, intracellular membrane traffic, modulation of surface receptors, and the mitosis process [41]. By phalloidin staining, the influence of the RKKP coated support on hAMSC adhesion, flattening, and lengthening was studied visualizing the actin cytoskeleton morphology and organization. It can be noticed that human mesenchymal stem cells attached on RKKP coated Ti surface maintain their morphology, shape, size, and orientation, similar to the cells grown on treated plastic Petri dishes, allowing the attachment and the actin cytoskeleton organization (Figure 5).

For further testing of cell-friendly and biocompatible behavior, we also analysed in the placenta derived mesenchymal stem cells grown on RKKP coated Ti surface the DNA synthesis through cell cycle phase distribution and performed an immunophenotypical characterization by flow cytometry analysis.

The propidium iodide labelled DNA showed no statistically significant differences in the cell phase percentage of hAMSCs grown on RKKP coated Ti surface and on the treated plastic Petri dishes, indicating that in these cells the cell cycle progression is not inhibited (Figure 6(a)). We also detected a high mesenchymal CD29 and CD73 stem cell markers' expression, whereas the hematopoietic CD31 stem cell marker was undetected in both samples (Figure 6(b)).

To confirm the RKKP supports biocompatibility and stem cell-friendliness, we analysed the health of hAMSCs studying the mRNAs' expression necessary to understand if their biosynthetic machine works properly even at transcriptional level.

With this aim, the mRNA expressions of placenta derived mesenchymal stem cell key genes, such as anti-inflammatory and trophic factors as well as the constitutive gene, were studied and in both samples the gene expressions resulted in being unchanged (Figure 7).

More importantly, we also verified the osteogenic commitment capability of the hAMSCs grown on the RKKP coated Ti surface and on the treated plastic Petri dish substrates, as a valuable indicator of their health and consequently of their capability of maintaining their differentiation potential.

Our results highlight that the chemically induced differentiation process is efficiently activated in the cells grown on the RKKP substrate like on the treated plastic Petri dish, and they expressed early and late bone differentiation markers, a clear indicator of their health and materials' biocompatibility (Figure 8).

In clinical applications, the RKKP glass-ceramic should be taken into account for its capability of controlled trace element release involved in the biochemical cycles of the human body. The choice of the trace elements to be released by the specific RKKP composition, guided by the type of application, depends on the living tissue to regenerate and the specific cellular response needed [20, 42]. Some biomaterials, although exhibiting good osteointegration performance in normal bone tissue, result in being unable to perform this process in osteopenic bone tissue.

As a matter of fact, in pathological bone tissue the success of the implant of a device is difficult to achieve for the absence of specific molecules managing specific biochemical cycles and especially for the effects due to the interaction of the bone with potentially toxic materials that might interfere with cell growth. In this context instead, RKKP glass-ceramic exhibits a higher osteointegration rate compared to other ceramic materials when implanted in the osteopenic bone [42, 43]. This difference can be explained by the chemical-physical characteristics of RKKP glass-ceramic, which is influenced by the local microenvironment of the bone site and it responds with an ionic release, differently from the one shown in the case of implants in the healthy bone [42, 44]. The different ionic exchange observed in the RKKP glass-ceramic confirms the existence of different biochemical mechanisms, intrinsic in healthy and osteopenic bones, that influence the dissolution, precipitation, and ion exchange reactions and consequently the osteointegration processes of this glass material [45], an important issue to be taken into due account by surgeons.

## 5. Conclusion

In conclusion, all these reasons, together with the anti-inflammatory, immunomodulatory, proangiogenic, and proregenerative properties, ideal intrinsic features of the placenta derived mesenchymal stem cells, render our cellularized RKKP coated Ti surface a very interesting cell delivery system that could be considered in the planning of regenerative medicine protocols and then used successfully for tissue engineering applications.

## Supplementary Material

Exponentially growing cells were seeded both on the RKKP coated Ti surface and on the plastic Petri dish and cultured up to 4 days. BrdU and WST-1 were added to the medium and absorbance of supernatant was measured according to the protocol described in the Supplementary Materials section.

## Figures and Tables

**Table 1:** Sequence of primers used for qRT-PCR.

Target gene	Primer sequence	Annealing temperature (C°)
VEGF	5′-cttgggtgcattggagcct-3′ 5′-ctgcgctgatagacatccat-3′	60
*β*-ACT	5′-gctcctcctgagcgcaag-3′ 5′catctgctggaaggtggaca-3′	60
Ki67	5′-tgaacaaaaggcaaagaagac-3′ 5′-gagctttccctattattatggt-3′	60
IDO	5′-tgctaaaggcgctgttggaa-3′ 5′-tacaccagaccgtctgatag-3′	60
HGF	5′-caatagcatgtcaagtggag-3′ 5′-ctgtgttcgtgtggtatcat-3′	60
TGF *β*1	5′-tcaagttaaaagtggagcagc-3′ 5′-actccggtgacatcaaaaga-3′	60
RPL34	5′-gaaacatgtcagcagggcc-3′ 5′-tgactctgtgcttgtgcctt-3′	60
RUNX2	5′-catcatctctgccccctct-3′ 5′-actcttgcctcgtccactc-3′	60
ALP	5′-caatgagggcaccgtggg-3′ 5′-tcgtggtggtcacaatgcc-3′	60
OCL	5′-gcagcgaggtagtgaagag-3′ 5′-gaaagccgatgtggtcagc-3′	60
GAPDH	5′-catcatctctgccccctct-3′ 5′-caaagttgtcatggatgacct-3′	60

**Table 2:** SEM-EDS elemental analysis results for RKKP bulk and film.

Spectrum/element	O	F	Na	Mg	Si	P	K	Ca	La	Ta	Total
RKKP bulk	45.0	5.2	2.7	1.5	18.2	3.7	0.2	19.6	0.8	3.1	100.0
Film	45.6	6.3	4.2	2.4	18.7	3.5	0.2	16.9	0.4	1.8	100.0

All results are in weight%.
