# Supplementary material for: Placenta Derived Mesenchymal Stem Cells Hosted on RKKP Glass-Ceramic: A Tissue Engineering Strategy for Bone Regenerative Medicine Applications
Source: Biomed Res Int. 2016 Dec 19;2016:3657906. doi: 10.1155/2016/3657906 (PMC5204102; doi:10.1155/2016/3657906)
Supplement: Supplementary file 1 — Exponentially growing cells were seeded both on the RKKP coated Ti surface and on the plastic Petri dish and cultured up to 4 days. BrdU and WST-1 were added to the medium and absorbance of supernatant was measured according to the protocol described in the Supplementary Materials section. [file 3657906.f1.pdf]

Supplementary information

Materials and methods

hAMSC isolation and culture

Homogenous hAMSCs populations were obtained by a two-step procedure. Small pieces of amniotic membrane were treated for 1 hour with 0.25% trypsin-EDTA solution to remove human amniotic epithelial cells (hAEC). The supernatant was discarded, the amnion pieces were carefully washed in phosphate-buffered saline (PBS) and subsequently digested with 0.1% collagenase IV (Sigma-Aldrich, St. Louis, USA), 20 µg/ml DNAse I (Sigma-Aldrich,) solution in Dulbecco's modified Eagle's medium (DMEM) for 2-3 hours [1]. The supernatant was transferred to fresh tubes and the enzymes were neutralized with Fetal Bovine Serum (FBS). The cells were spun down at 300 x g for 10 minutes and the pellet was suspended in DMEM supplemented with 10% FBS, penicillin (100 U/ml), streptomycin (100 µg/ml) (PBI international Milano, Italy) and Epidermal Growth Factor (EGF) (10ng/ml, ImmunoTools, Friesoythe, Germany). hAMSCs were cultured on the tissue-treated Petri dish and maintained at 37°C in a humidified atmosphere containing 5% CO_2. Non-adherent cells were removed after 1 week and, when the culture reached 90% confluence, hAMSCs were harvested using 0.25% Trypsin-EDTA and sub-cultured at a density of 1×10^4 cells/cm^2 in the plastic Petri dish. The medium was subsequently changed every 3 days.

Cell proliferation and metabolic activity analysis

Exponentially growing cells were seeded both on the RKKP coated Ti surface and on the plastic Petri dish at a density of $1x10^4$ cells/cm^2 and cultured up to 4 days. 10 mM Bromodeoxyuridine was added to the medium at day 1, 2, 3 and 4 after plating and maintained for 18 h in culture. Cells were then fixed and incubated for 30 min at room temperature with the anti-BrdU antibody (1:100; Cell Proliferation Kit; Roche Diagnostics). After incubation with 2,20-Azino-bis (3-ethylbenzothiazoline-6-sulfonic acid) for 30 min, the absorbance of 100µl of supernatant was measured in an ELISA reader (VICTOR3 multilabel readers; PerkinElmer, Waltham, Massachusetts) at 450 nm.

Exponentially growing hAMSCs were seeded both on the RKKP film surface and on the plastic Petri dish at a density of $1x10^4$ cells/cm^2, and cultured up to 4 days in a humidified incubator (37°C, 5% CO_2). WST-1 reagent was added to the cell medium at a dilution of 1:10 at day 1, 2, 3 and 4, following plating. After 2 hours of

incubation in a humidified atmosphere, 100 μl supernatant was transferred in 96-well plates and analysed by means of formazan dye. Quantification of the produced formazan dye was performed measuring the absorbance at 450 nm with an ELISA reader (VICTOR3 multilabel readers; PerkinElmer, Waltham, Massachusetts).

Real-Time quantitative RT-PCR analysis

Experiments were conducted to contrast relative levels of each transcript and endogenous control Glyceraldehyde 3-phosphate dehydrogenase (GAPDH) in every sample. Gene expression was presented using the (2^{-DDCt}) method, described by Livak and Schmittgen [2], where DCt = (average target Ct – average GAPDH Ct), DDCt = (average DCt treated sample – average DCt untreated sample). We performed a validation experiment to prove that the amplification efficiency of target genes and reference GAPDH was equal. RT-PCR was performed with Sybr Green I Mastermix, using an ABI PRISM 7000 Sequence Detection System (Applied Biosystems, Foster City, CA, USA). Each reaction was run in triplicate and contained 0.5–1 μL of cDNA template, with 250 nM primers in a final reaction volume of 25 μL. The specific primers and annealing temperatures used are reported in Table S1. The annealing temperature used for all primers in this study is 60°C. Cycling parameters were: 50°C for 2 min, 95°C for 10 min the (to activate DNA polymerase), then 40–45 cycles at 95°C for 15 s and 60°C for 1 min. Melting curves were performed using Dissociation Curves software (Applied Biosystems) to ensure that only a single product had been amplified. As negative controls, reactions were prepared, in which RNA or reverse transcriptase had previously been omitted during reverse transcription.

[1] Casey M L and MacDonald P C 1996 Interstitial collagen synthesis and processing in human amnion: a property of the mesenchymal cells *Biol. reprod.* **55** 1253-60

[2] Livak K J and Schmittgen T D 2001 Analysis of relative gene expression data using real-time quantitative PCR and the $2^{-\Delta\Delta CT}$ method *Methods* **25** 402-8

Table S1. Sequence of primer used for qRT-PCR

Target gene	Primer sequence	Annealing temperature (C°)
VEGF	5’-cttgggtgcattggagcct-3'<br>5’-ctgcgctgatagacatccat-3'	60
β-ACT	5'-gctcctcctgagcgcaag-3'<br>5'catctgctggaaggtggaca-3'	60
Ki67	5'-tgaacaaaaggcaaagaagac-3'<br>5'-gagctttccctattattatggt-3'	60
IDO	5’-tgctaaaggcgctgttggaa-3’<br>5’-tacaccagaccgtctgatag-3’	60
HGF	5’-caatagcatgtcaagtggag-3’<br>5’-ctgtgttcgtgtggtatcat -3’	60
TGF β1	5’-tcaagttaaaagtggagcagc-3’<br>5’-actccggtgacatcaaaaga-3’	60
RPL34	5’-gaaacatgtcagcagggcc-3’<br>5’-tgactctgtgcttgtgcctt-3’	60
RUNX2	5'-catcatctctgccccctct-3'<br>5'-actcttgcctcgtccactc-3'	60
ALP	5'-caatgagggcaccgtggg-3'<br>5'-tcgtggtggtcacaatgcc-3'	60
OCL	5'-gcagcgaggtagtgaagag-3'<br>5'-gaaagccgatgtggtcagc-3'	60
GAPDH	5'-catcatctctgccccctct-3'<br>5'-caaagttgtcatggatgacct-3'	60
